# The Effects of Gut Microbiota, Plasma Metabolites, Immune Cells, Blood Cells and Cytokines on Schizophrenia: A Bidirectional Two-Sample Mendelian Randomisation Study

**DOI:** 10.62641/aep.v54i1.2014

**Published:** 2026-02-15

**Authors:** Kaihong Zhang, Wen Sun, Qingxiang Meng, Gaowei Liu, Xinyuan Hu, Sufang Qi, Xi Liu, Wei Wang, Junmei Wei, Jiawei Jin, Cuicui Zhang, Yu Cao

**Affiliations:** ^1^Department of Psychiatry, Shandong Mental Health Center, 250014 Jinan, Shandong, China

**Keywords:** schizophrenia, mendelian randomisation, mediation analysis, gut microbiota, plasma metabolites

## Abstract

**Background:**

The aberrant traits of the gut–metabolite–immune network in schizophrenia imply a crucial interrelationship among them. The exploration of the association between the gut–metabolite–immune network and schizophrenia will create novel opportunities for future studies on the disorder.

**Methods:**

This study utilised the Mendelian randomisation (MR) method to examine the causal relationships among gut microbiota, plasma metabolites, immune cells, blood cells, cytokines and schizophrenia. Additionally, mediation analysis was performed to identify and verify potential mediators involved in the pathway linking gut microbiota to schizophrenia.

**Results:**

A total of 62 traits with causal connections to schizophrenia were identified from the gut microbiota, plasma metabolites, immune cells, blood cells and cytokines (11 traits from the gut microbiota [odds ratio (OR) = 0.683–2.104, *p* = 0.005–0.047], 35 traits from plasma metabolites [OR = 0.596–1.597, *p* = 0.005–0.049], 14 traits from immune cells [OR = 0.813–1.105, *p* = 0.005–0.049], 1 trait from blood cells [OR = 1.112, *p* = 0.038] and 1 trait from cytokines [OR = 0.864, *p* = 0.041]). Among them, 30 traits were classified as risk factors for schizophrenia. Additionally, we determined nine pathways by which gut microbiota influences schizophrenia (via 7 plasma metabolites and 2 immune cells). Moreover, in our MR analyses, several sensitivity analyses were employed to eliminate heterogeneity and horizontal pleiotropy, ensuring reliable MR results. Meanwhile, the outcomes of various analyses revealed that the gut microbiota most significantly associated with schizophrenia belonged to the Firmicutes phylum.

**Conclusions:**

These discoveries not only deepen our understanding of the pathogenic mechanism of schizophrenia but also offer significant impetus for the development of future diagnostic studies and therapeutic strategies.

## Introduction

Schizophrenia (SCZ) is a prevalent neurological disorder often emerging during young adulthood to middle age, and its precise aetiology remains unclear. SCZ hinders patients’ social interactions and formation of interpersonal relationships by causing deficits in various domains, such as cognition, perception, emotion, volition, behaviour and cognitive function [1, 2]. In particular, cognitive dysfunction is regarded as the principal characteristic of SCZ and is the primary factor contributing to the long-term disability related to the disorder [3, 4]. Numerous previous studies have demonstrated that cognitive function is regulated and influenced by multiple factors including the gut microbiota and its metabolites [5, 6] and immune-inflammatory responses [7].

Numerous studies have indicated that the gut–brain axis plays a crucial role in the pathogenesis of mental disorders. The complex interactions among the gut microbiota, the nervous system and the immune system significantly influence an individual’s psychological state and mood, and they are involved in the regulation of hormone levels [8]. Patients with SCZ exhibit significant disorders of the gut microbiota [9, 10] and dysregulation of short-chain fatty acid (SCFA) production [11]. Imbalances in gut microecology may interfere with the regular functions of the nervous system, thereby exerting adverse effects on brain function [12].

Similarly, the imbalance in microbial metabolite levels has a remarkable influence on the brain function in SCZ and is closely related to immune regulatory functions. A multi-omics study has conducted comparative analyses of gut microbiota and serum metabolites between patients with SCZ and healthy individuals. The results revealed a significant increase in the content of pro-inflammatory metabolites but a significant decrease in the content of anti-inflammatory metabolites [13]. Furthermore, some studies have indicated that the levels of glutamate metabolites and γ-aminobutyric acid (GABA) may be associated with the pathophysiological mechanisms of SCZ spectrum disorders [14]. Further research also indicated that the neurotransmission pathways of GABA and tryptophan are related to the risk of SCZ, among which GABA may play a more crucial role than tryptophan [15].

The irregularities in the gut–metabolite–immune network in SCZ imply that the disorder of the immune–inflammatory pathway may be involved in the pathophysiological mechanisms of SCZ spectrum disorders. Several studies have demonstrated that the abnormal expression of inflammatory factors can aggravate the symptoms of SCZ. Specifically, the levels of certain inflammatory factors, such as interleukin-6 (IL-6) [16], tumour necrosis factor-α (TNF-α) [17], interleukin-2 receptor (IL-2R) [18], interleukin-17 (IL-17) [19] and chemokines CCL-2 and CXCL-8 [20], in patients with SCZ are significantly higher than those in healthy individuals. Furthermore, the phenotypes and clinical manifestations of SCZ are accompanied with an elevated concentration of acute-phase proteins, including transferrin and the complement system [21, 22]. Simultaneously, immune cells with pro-inflammatory or regulatory functions in SCZ, such as M1 macrophages [23], Th-1 cells [24] and regulatory T cells [25], exhibit signs of abnormal activation.

To comprehensively elucidate the causal relationships among gut microbiota, plasma metabolites, immune cells, blood cells, cytokines and SCZ within the gut–brain axis, and to validate whether the pathway from gut microbiota to SCZ is mediated by plasma metabolites, immune cells, blood cells and cytokines, this study employed the Mendelian randomisation (MR) approach to conduct an in-depth analysis of publicly available genome-wide association study (GWAS) summary data. MR, as a statistical tool grounded in genetic variations, facilitates the accurate investigation of causal effects between exposure factors and outcomes by introducing instrumental variables (IVs) [26]. Notably, within the MR framework, the direction of causal relationships indicated by genetic associations is clear. Genetic variations are the source, not the consequence, of phenotypic differences. This unique feature enables researchers to infer causal relationships between exposure factors and outcomes with enhanced accuracy [27]. Mediation MR utilises genetic variations as IVs. Through multivariable MR or two-step MR approaches, it decomposes the direct effect of an exposure on an outcome and the indirect effect via mediating variables. This serves to control confounding factors and validate causal pathways [28]. In the research of the brain–gut axis, this method can analyse how mediating factors such as plasma metabolites mediate the causal mechanisms of brain diseases (e.g., Alzheimer’s disease). For example, it can identify the indirect effects of specific microbiota on neural function through immune or metabolic pathways [29, 30]. In the present study, mediation MR was utilised to evaluate the causal pathways from gut microbiota to plasma metabolites, blood cells, immune cells, cytokines and SCZ mediated by these factors.

## Materials and Methods

### Study Design

**Fig. 1.**
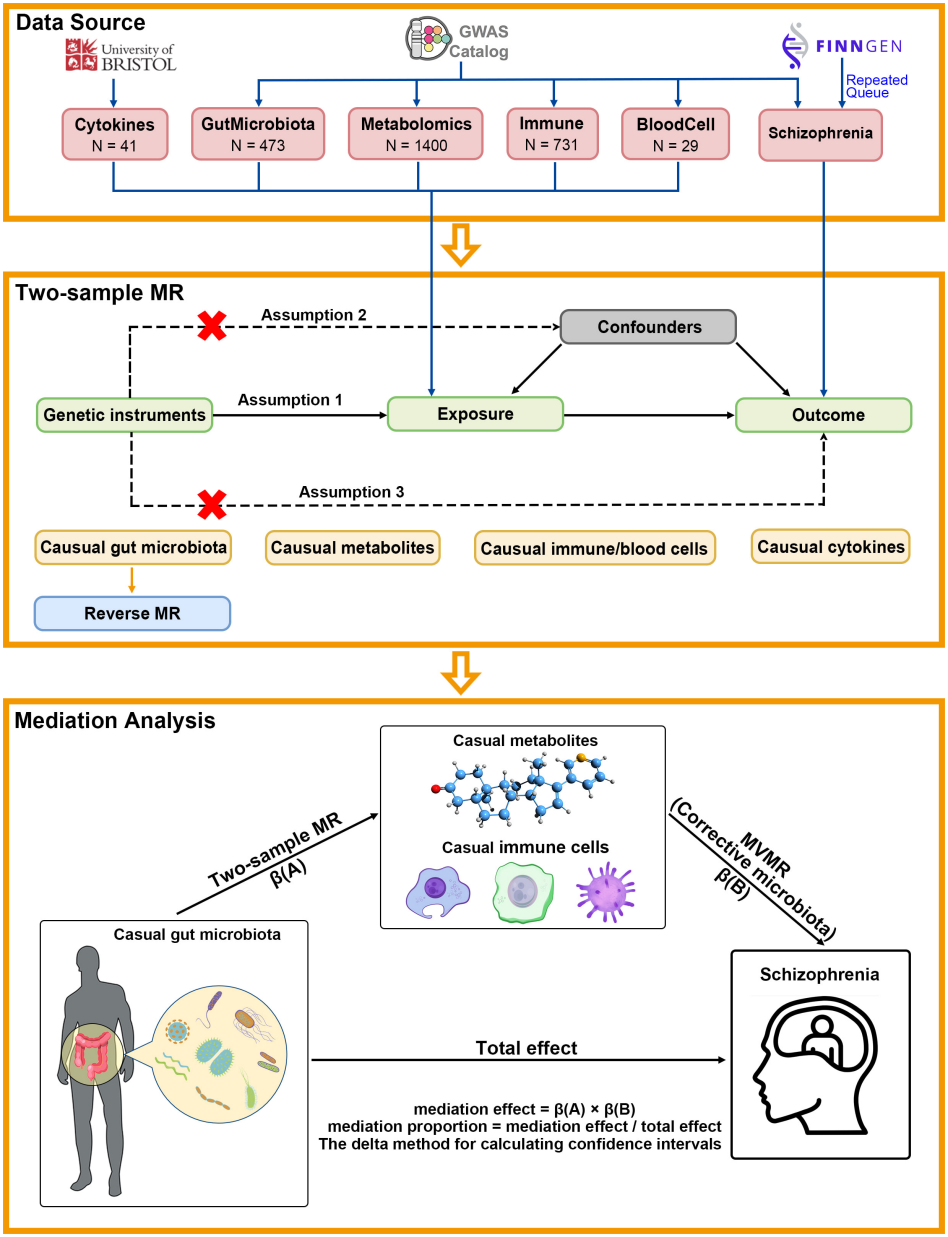
**Flowchart of the study**. The association hypothesis, independence hypothesis and exclusion hypothesis of bidirectional two-sample Mendelian randomisation. The direct effect, indirect effect and total effect in the mediation analysis are presented. Within the analytical framework of two-sample Mendelian randomisation, this study designated gut microbiota, plasma metabolites, blood cells, immune cells and cytokines as exposure variables and schizophrenia as the core outcome variable. The aim was to systematically investigate the potential causal associations between these exposure factors and schizophrenia. In the subsequent mediation effect analysis, the research design was further refined. Specifically, gut microbiota was still considered the exposure variable, and schizophrenia was the outcome variable. Meanwhile, the mediator variables were confined to plasma metabolites and immune cells. These two variables had been validated through two-sample Mendelian randomisation, and they were established to have causal relationships with schizophrenia and microbiota. This approach was employed to uncover the possible metabolic-immune mediating pathways through which gut microbiota influences the pathogenesis of schizophrenia.

The flowchart for this study is presented in Fig. 1. We initially collected the published summary data of GWAS from the GWAS Catalog database, which encompassed traits such as the gut microbiota, plasma metabolites, immune cells, blood cells and cytokines. These data are summarised in **Supplementary Table 1–S1**. Subsequently, we carried out bidirectional two-sample MR analyses among these traits and SCZ to assess the causal relationships between them. Finally, to determine the mediating roles of plasma metabolites and other traits on the association between the gut microbiota and SCZ, we conducted MR analyses based on a two-step approach and multivariable MR (MVMR) analysis. The MR analyses in our study were designed in accordance with the STROBE-MR guidelines [31].

### Data Sources

All the GWAS summary statistics employed in this study were derived from the GWAS Catalog (https://www.ebi.ac.uk/gwas/), the University of Bristol (https://research-information.bris.ac.uk/en/datasets/) and FinnGen (https://www.finngen.fi/en/access_results).

The GWAS statistical data on gut microbiota were obtained under the accession numbers GCST90032172-GCST90032644 of the GWAS Catalog. The data included microbial classifications from 5959 individuals from Europe (Finland), which comprised 11 phyla, 19 classes, 24 orders, 62 families, 146 genera and 209 species [32]. In this study, we used the GWAS summary data of 473 gut microbial components for subsequent MR analyses. The GWAS data collected from FinnGen (with the accession number KRA_PSY_SCHIZODEL_EXMORE) served as our replicated cohort in this research, aiming to validate the accuracy of the analysis results.

The GWAS statistical data of the plasma metabolomics originated from the research cohort of the GWAS Catalog with the accession numbers GCST90199621-GCST90204063. This dataset encompassed 1091 metabolites and 309 metabolite ratios from 8299 individuals in Canada. Among them, 850 metabolites had clearly defined and known roles in the 8 metabolic groups: lipid, amino acid, xenobiotics, nucleotide, cofactor and vitamins, carbohydrate, peptide and energy [33].

The summary statistics of immune cells (Immune) were obtained by downloading GCST0001391-GCST0002121 from the GWAS Catalog. These data were derived from the peripheral blood of 6602 individuals, measured via flow cytometry and analysed to evaluate a total of 731 cell traits, 192 relative counts, 389 MFIs of surface antigens and 32 morphological parameters [34] (accession numbers for each trait are summarised in **Supplementary Table 1–S1**).

The GWAS data associated with blood cells (BloodCell) were retrieved through the accession number GCST90002379-GCST90002407 of the GWAS Catalog. This dataset comprised 563,085 participants of European ancestry and disclosed 5106 novel variants that were independently associated with 29 blood cell phenotypes and jointly influenced the hematopoietic process [35] (accession numbers for each trait are summarised in **Supplementary Table 1–S1**).

The GWAS data regarding cytokines originated from the University of Bristol, with the accession number of 3g3i5smgghp0s2uvm1doflkx9x. This dataset encompassed the genetic information of 8293 Finns and mainly explored the genetic basis underlying the circulating levels of 41 cytokines [36].

The disease research cohort containing SCZ from the GWAS Catalog, with the accession number of GCST90018919, comprised 6334 cases of European ancestry, 445,120 controls of European ancestry, 99 cases of East Asian ancestry and 177,794 controls of East Asian ancestry. Moreover, this study summarises 47 disease states and creates individual-level phenotypes for 159 disease endpoints [37].

### IV Selection

IVs directly influence the accuracy and reliability of causal inference and must satisfy the assumptions of association, independence and exclusion: (1) There must be a significant association between IVs and the exposure factor. (2) IVs need to be independent of confounding factors, that is, they can only indirectly affect the outcome by acting on the exposure factor rather than through any other means. (3) The influence of IVs on the outcome must be strictly transmitted through the exposure factor, excluding any other possible direct paths. According to these principles, the IVs used in this study were filtered through the following conditions: (1) the selection of the genome-wide significance threshold *p* < 5 × 10^-8^ (if the number of SNPs was less than 5, filtering was performed using *p* < 5 × 10^-5^); (2) retention of SNPs that were not related to the outcome variable (*p*val.outcome > 0.05); (3) removal of SNPs with linkage disequilibrium (LD) to ensure independence among IVs (cytokines, gut microbiota, immune and metabolomics: window size = 500 kb, r^2^ < 0.01; blood cells: window size = 10,000 kb, r^2^ < 0.001); (4) removal of weak IVs that could not provide sufficient statistical power [38], using the F statistic to assess the strength of the selected SNPs and retaining SNPs with F >10; (5) application of the MR residual and outlier (MR-PRESSO) test [39] to evaluate heterogeneity at the MR level by identifying and correcting outliers to reduce their influence on causal inference (MR-PRESSO global test *p* > 0.05); and (6) use of the Harmonise and Steiger methods to calculate the proportion of explanation of IVs for the exposure factor and outcome variable to filter out IVs with incorrect causal relationship directionality (that is, the proportion of explanation for the outcome variable was greater than that for the exposure factor).

### Two-Sample Mendelian Randomisation (TSMR)

The TSMR approach was employed to assess the causal relationships among these traits and SCZ. The Wald ratio and inverse variance weighted (IVW) were utilised to infer the causal association of exposure. Among them, IVW has emerged as the preferred and commonly adopted method in MR analysis because of its strong estimation effect and ability to enhance estimation accuracy. The Wald ratio is mainly applied to estimate the effect size of individual SNPs for inferring the causal relationship between exposure and outcome [40].

To evaluate the stability and reliability of causal relationships, we performed a series of sensitivity analyses in this study. MR-PRESSO was employed to detect horizontal pleiotropy in MR analysis and reduce the influence of horizontal pleiotropy on causal inference by adjusting outliers. The determination of the causal direction in MR analysis was verified by the Steiger method. If the association of IVs with the exposure factor was greater than that with the outcome variable, then the directionality might be correct, that is, the exposure factor might lead to the outcome variable.

All the MR analyses in this study were carried out through R software (version 4.3.1, R Foundation for Statistical Computing, Vienna, Vienna State, Austria). The bidirectional two-sample MR analysis was implemented using the R package ‘TwoSampleMR’ (version 0.6.7, MRC Integrative Epidemiology Unit, Bristol, England, United Kingdom) (https://github.com/MRCIEU/TwoSampleMR), and multivariable MR analysis was conducted with the R package ‘MendelianRandomization’ (version 0.9.0, Comprehensive R Archive Network, Vienna, Vienna State, Austria) (https://github.com/cran/MendelianRandomization). All MR analyses were subjected to the test for horizontal pleiotropy via the R package MR-PRESSO (version 1.0, Ron Do Lab, Boston, MA, USA) (https://github.com/rondolab/MR-PRESSO).

Considering the potential risk of inflated overall type I errors during multiple comparisons, to control the false positive rate in the process of statistical inference, this study employed the Benjamini–Hochberg correction procedure to adjust for FDR on the main results of IVW analysis. When the FDR value was less than 0.1, the corresponding association was considered statistically significant.

### Reverse MR Analysis

To investigate whether SCZ exerts a causal effect on the identified gut microbiota, we conducted a reverse MR analysis. In this approach, SNPs associated with SCZ were selected as IVs based on the following criteria: *p* < 10^-8^, window size of 10,000 kb, LD r^2^ < 0.001 and F-statistic >10. Here, SCZ was treated as the exposure, whereas the gut microbiota and other traits were designated as outcome traits. The analytical framework for reverse MR followed the same methodological principles as the primary MR analysis.

### Mediation Analysis

Mediator analysis facilitates the elucidation of the internal mechanisms underlying the influential relationship between exposure factors and outcome variables. It explores whether the influence of exposure factors on outcome variables occurs through a certain variable. If the exposure factors affect the outcome variables by influencing this variable, then this variable is termed as the mediator variable [41]. The mediator effect model encompasses three types of effects: (1) direct effect (the direct influence of exposure factors on outcome variables); (2) indirect effect (the indirect influence of exposure factors on outcome variables through the mediator variable, namely, the path effect of exposure factors → mediator variable → outcome variables); and (3) total effect (the overall influence of exposure factors on outcome variables, including direct and indirect effects).

This study focused its mediation analysis on the interplay among gut microbiota, plasma metabolites, immune cells, blood cells, cytokines and SCZ. Initially, TSMR was used to estimate the causal effect β(A) of gut microbiota on plasma metabolites, immune cells, blood cells and cytokines. Subsequently, MVMR was applied to identify those plasma metabolites, immune cells, blood cells and cytokines that maintained a significant causal association with SCZ after accounting for the influence of gut microbiota, thereby quantifying the adjusted causal effect β(B). Finally, the mediation effect was computed using two-step MR: mediation effect = β(A) × β(B). The direct effect was calculated as follows: direct effect = (total effect – mediation effect). The mediation proportion was calculated as follows: mediation proportion = mediation effect / total effect. The Delta method was utilised to estimate the 95% confidence intervals (CIs) for the mediation effect and the mediated proportion. The two-sided method was employed to calculate the *p* value. For all statistical tests, a two-sided *p* value less than 0.1 was regarded as statistically significant. If the direct effect of the exposure factor on the outcome variable was not significant but the indirect effect was significant, then the mediating variable exerted a complete mediating role between the exposure factor and the outcome variable. If the direct and indirect effects of the exposure factor on the outcome variable were significant, then the mediating variable played a mediating role between the exposure factor and the outcome variable [42].

## Results

### Causal Influence of Gut Microbiota on SCZ

**Fig. 2.**
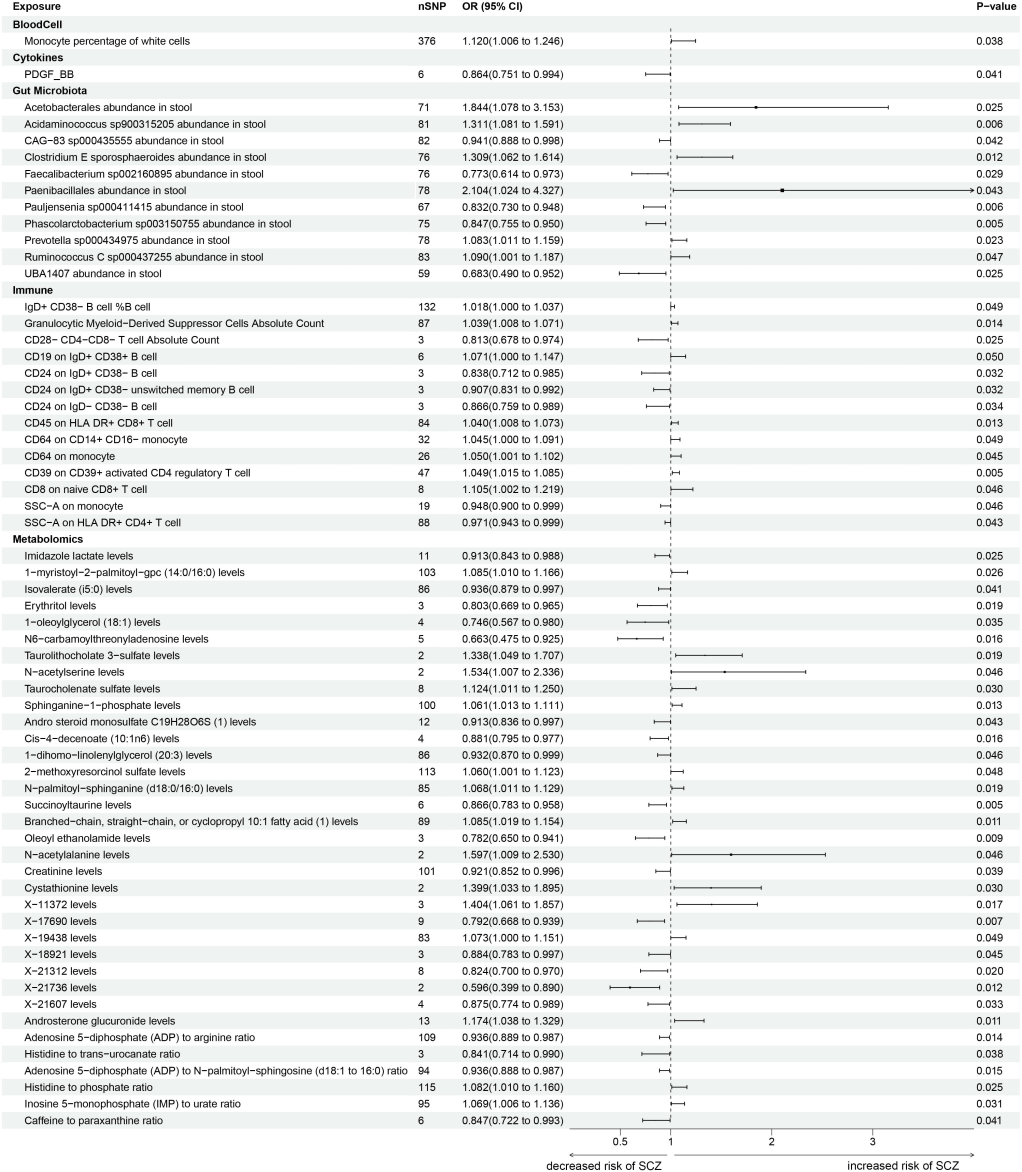
**Forest plots illustrating the causal associations among gut microbiota, plasma metabolites, immune cells, blood cells, cytokines and schizophrenia**. The horizontal lines along the x-axis depict the 95% confidence intervals (CIs) for the estimated causal effects. All associations displayed in this figure are statistically significant. nSNP denotes the number of single nucleotide polymorphisms (SNPs); OR denotes odds ratio; CI denotes confidence interval.

The brain–gut axis plays a crucial role in the pathogenesis of SCZ. We performed causal analysis on the GWAS data of the intestinal microbiota and SCZ through two-sample MR and obtained 11 suggestive associations (*p*_IVW_ < 0.05) between the intestinal microbiota and SCZ (Fig. 2) (**Supplementary Table 1–S2**). *Acetobacterales*, *Acidaminococcus* sp900315205, *Clostridium* E sporosphaeroides, *Paenibacillales*, *Prevotella* sp000434975 and *Ruminococcus* C sp000437255 showed a positive causal relationship with SCZ. Among them, the associations were particularly prominent for Acetobacterales (OR = 1.039, 95% CI = 1.008–1.071, *p* = 0.014) and *Paenibacillales* (OR = 1.039, 95% CI = 1.008–1.071, *p* = 0.014). CAG-83 sp000435555, *Faecalibacterium* sp002160895, *Pauljensenia* sp000411415, *phascolarctobacterium* sp003150755 and UBA1407 showed a negative causal relationship with SCZ. For this causal effect result, additional tests such as MR-Egger, MR-PRESSO and MR-Steiger were conducted for sensitivity analysis. We found no evidence of horizontal pleiotropy or reverse causality in the data, and Q statistics also indicated no heterogeneity in this result (**Supplementary Table 1–S3**).

Regarding the causal relationship between the gut microbiota and SCZ as described above, we performed a reverse MR analysis. The results indicated a positive causal association between SCZ and Mycobacteriaceae (OR = 1.021, 95% CI = 1.000–1.041, *p* = 0.040), as well as between SCZ and *Parabacteroides* (OR = 1.083, 95% CI = 1.000–1.172, *p* = 0.049) (**Supplementary Table 1–S4 and S5**).

### Causal Influence of Plasma Metabolites on SCZ

In the causal analysis of plasma metabolites and SCZ, we ultimately detected 29 metabolites and 6 metabolic ratios (Fig. 2) (**Supplementary Table 1–S6**). Among them, 15 metabolites exhibited a positive causal relationship with SCZ. N-Acetylalanine (OR = 1.597, 95% CI = 1.009–2.530, *p* = 0.0458) and N-acetylserine levels (OR = 1.534, 95% CI = 1.007–2.336, *p* = 0.0463) were the two traits with the most prominent positive causal relationships. Sensitivity analysis verified that our analysis results did not demonstrate horizontal pleiotropy and heterogeneity (**Supplementary Table 1–S7**).

### Causal Effects of Immune Cells, Blood Cells and Cytokines on SCZ

Among the 14 immune cell traits related to SCZ, 8 traits demonstrated a positive causal relationship with SCZ, among which the most prominent one was CD8 on naive CD8+ T cell (OR = 1.105, 95% CI = 1.002–1.219, *p* = 0.0458) (Fig. 2) (**Supplementary Table 1–S8 and S9**).

In the two-sample MR analysis, 1 trait associated with SCZ was identified for blood cells and cytokines (**Supplementary Table 1–S10**), namely, monocyte percentage of white cells (OR = 1.12, 95% CI = 1.006–1.246, *p* = 0.0376) and PDGF_BB (platelet-derived growth factor, OR = 0.864, 95% CI = 0.751–0.994, *p* = 0.0414). The above results also excluded the significant effects of horizontal pleiotropy and heterogeneity through sensitivity analysis (**Supplementary Table 1–S11**).

**Fig. 3.**
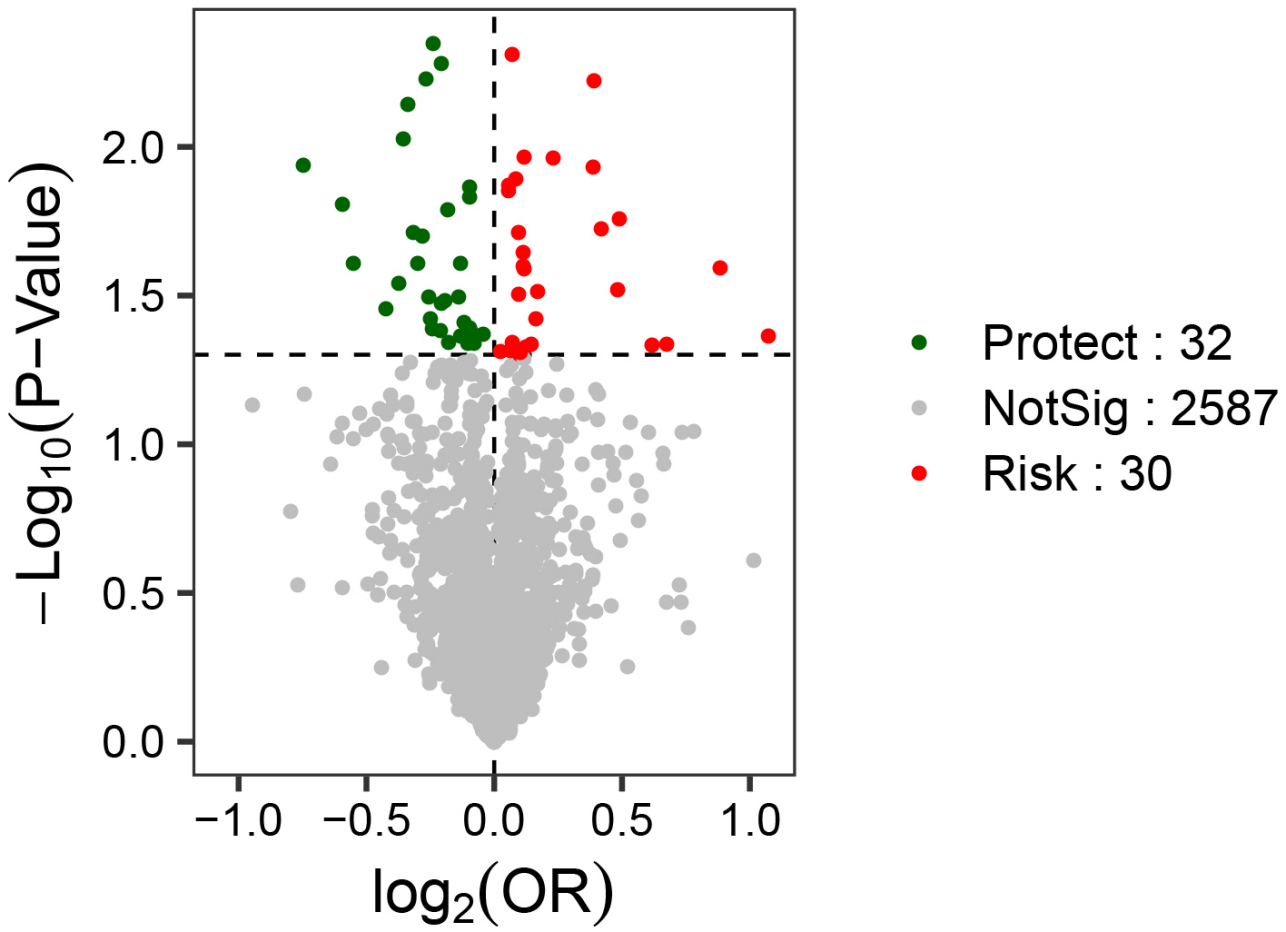
**Volcano plots of the causal associations among the gut microbiota, plasma metabolites, immune cells, blood cells, cytokines and schizophrenia**. The horizontal axis represents the magnitude of OR change (log_2_OR), and the vertical axis represents statistical significance (the log-transformed *p*).

On the basis of GWAS data among the gut microbiota, plasma metabolites, immune cells, blood cells, cytokines and SCZ, a total of 30 traits at risk for SCZ (Risk) and 32 traits protective against SCZ (Protect) were obtained in our two-sample MR analysis (Fig. 3).

### Results of Mediator Analysis

Our subsequent analysis aimed to explore the causal pathways from the gut microbiota to SCZ through mediator analysis, focusing on the mechanisms of its occurrence and development related to gut microbiota and other traits.

Firstly, the causal relationships among the gut microbiota and other traits were evaluated through two-sample MR. We identified 9 associations between the gut microbiota and plasma metabolites (including 4 gut microbiota traits and 9 plasma metabolite traits) and 2 associations between the gut microbiota and immune cells (including 2 gut microbiota traits and 2 immune cell traits) (**Supplementary Table 1–S12**). The results of this analysis were further demonstrated by sensitivity analysis to show no heterogeneity and horizontal pleiotropy (**Supplementary Table 1–S13**). MVMR analysis was employed to identify the plasma metabolites and immune cells that had a causal relationship with SCZ after adjusting for the gut microbiota. After adjusting for the gut microbiota, there were 7 plasma metabolite–SCZ associations and 2 immune cell–SCZ associations (**Supplementary Table 1–S14 and S15**).

In summary, we identified 9 mediating relationships between the gut microbiota and SCZ, including 7 causal pathways mediated by plasma metabolites and 2 causal pathways mediated by immune cells (Table 1) (**Supplementary Table 1–S16 and S17**). In this mediation analysis, 3 gut microbiota traits of CAG-83 sp000435555, *Clostridium* E sporosphaeroides and *Ruminococcus* C sp000437255 presented more than one mediating factor. Within all mediating relationships, the maximum mediating proportion reached 16.16%. However, in light of the findings of this study, the significance levels of these nine causal pathways were relatively low. For six causal pathways, the directions of the total effect, indirect effect and direct effect were all consistent.

**Table 1.** **Mediation effect of gut microbiota on schizophrenia via plasma metabolites and immune cells**.

Exposure	Mediator	Outcome	Total effect	Direct effect	Mediation effect (95% CI)	*p*-value	Mediation proportion
CAG-83 sp000435555	CD8 on naive CD8+ T cell	Schizophrenia	−0.060	−0.054	−0.006 (−0.013, 0.000)	0.067	10.41%
CAG-83 sp000435555	Caffeine to paraxanthine ratio	Schizophrenia	−0.060	−0.068	0.008 (−0.020, 0.035)	0.584	12.54%
Clostridium E sporosphaeroides	CD19 on IgD+ CD38+ B cell	Schizophrenia	0.269	0.254	0.016 (−0.027, 0.059)	0.479	5.78%
Clostridium E sporosphaeroides	Erythritol levels	Schizophrenia	0.269	0.231	0.039 (−0.014, 0.091)	0.146	14.36%
Clostridium E sporosphaeroides	X-17690 levels	Schizophrenia	0.269	0.226	0.044 (−0.008, 0.095)	0.096	16.16%
Faecalibacterium sp002160895	X-19438 levels	Schizophrenia	−0.258	−0.268	0.011 (−0.011, 0.033)	0.341	4.18%
Ruminococcus C sp000437255	Sphinganine-1-phosphate levels	Schizophrenia	0.086	0.090	−0.003 (−0.008, 0.001)	0.112	3.87%
Ruminococcus C sp000437255	Caffeine to paraxanthine ratio	Schizophrenia	0.086	0.076	0.010 (−0.017, 0.037)	0.475	11.49%
UBA1407	X-18921 levels	Schizophrenia	−0.382	−0.351	−0.031 (−0.076, 0.023)	0.185	8.08%

### Two-Sample MR and Mediation Analysis of Repeated Queues

**Fig. 4.**
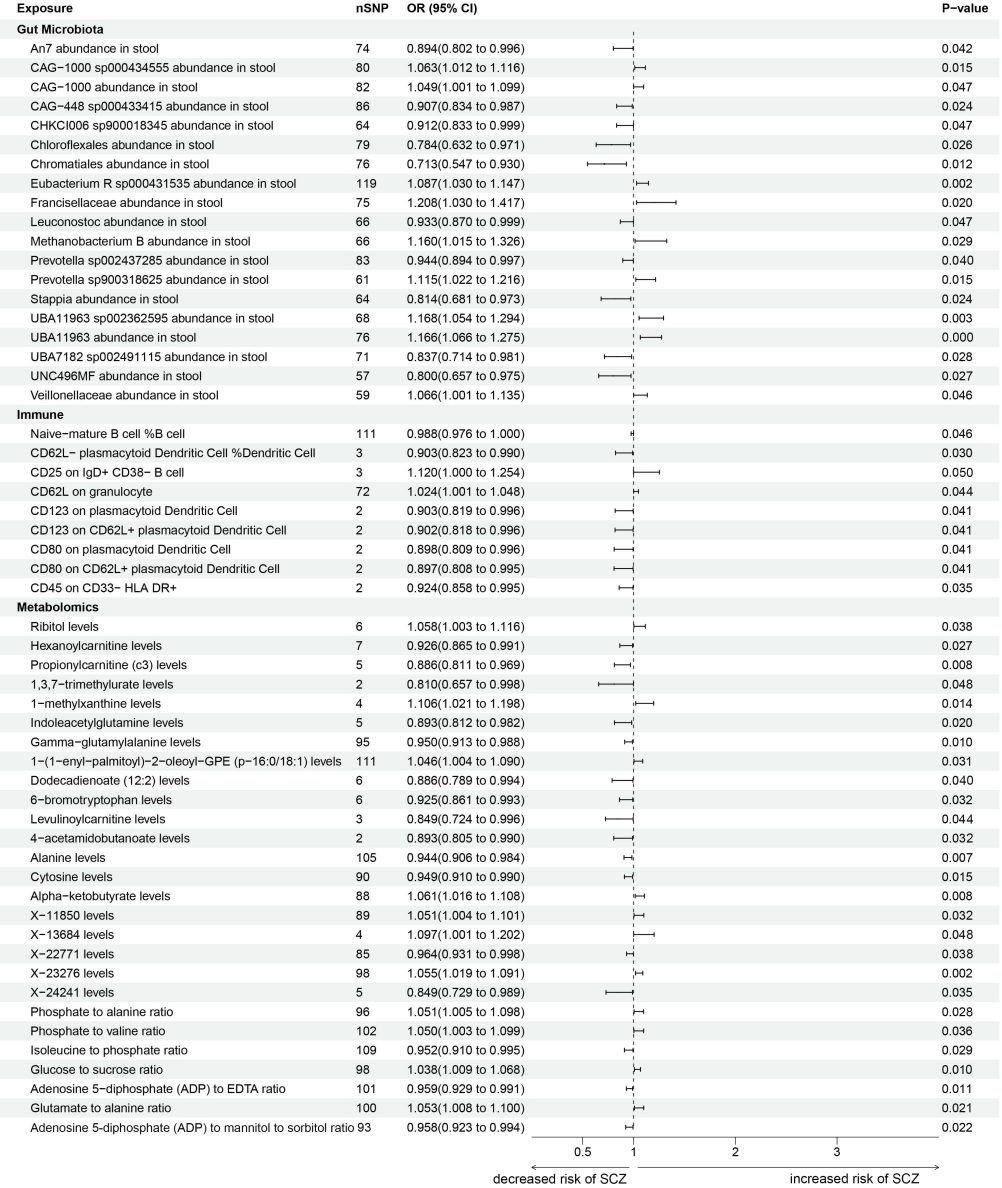
**Forest plots for causal effects among gut microbiota, plasma metabolites, immune cells, blood cells, cytokines and schizophrenia (repeat the queue analysis results)**. The horizontal bars on the abscissa represent the 95% confidence intervals obtained when estimating the causal effects of gut microbiota, plasma metabolites and immune cells. All the causal relationships shown in this figure exhibit statistical significance. nSNP denotes the number of single nucleotide polymorphisms (SNPs); OR represents odds ratio; CI represents confidence interval.

**Fig. 5.**
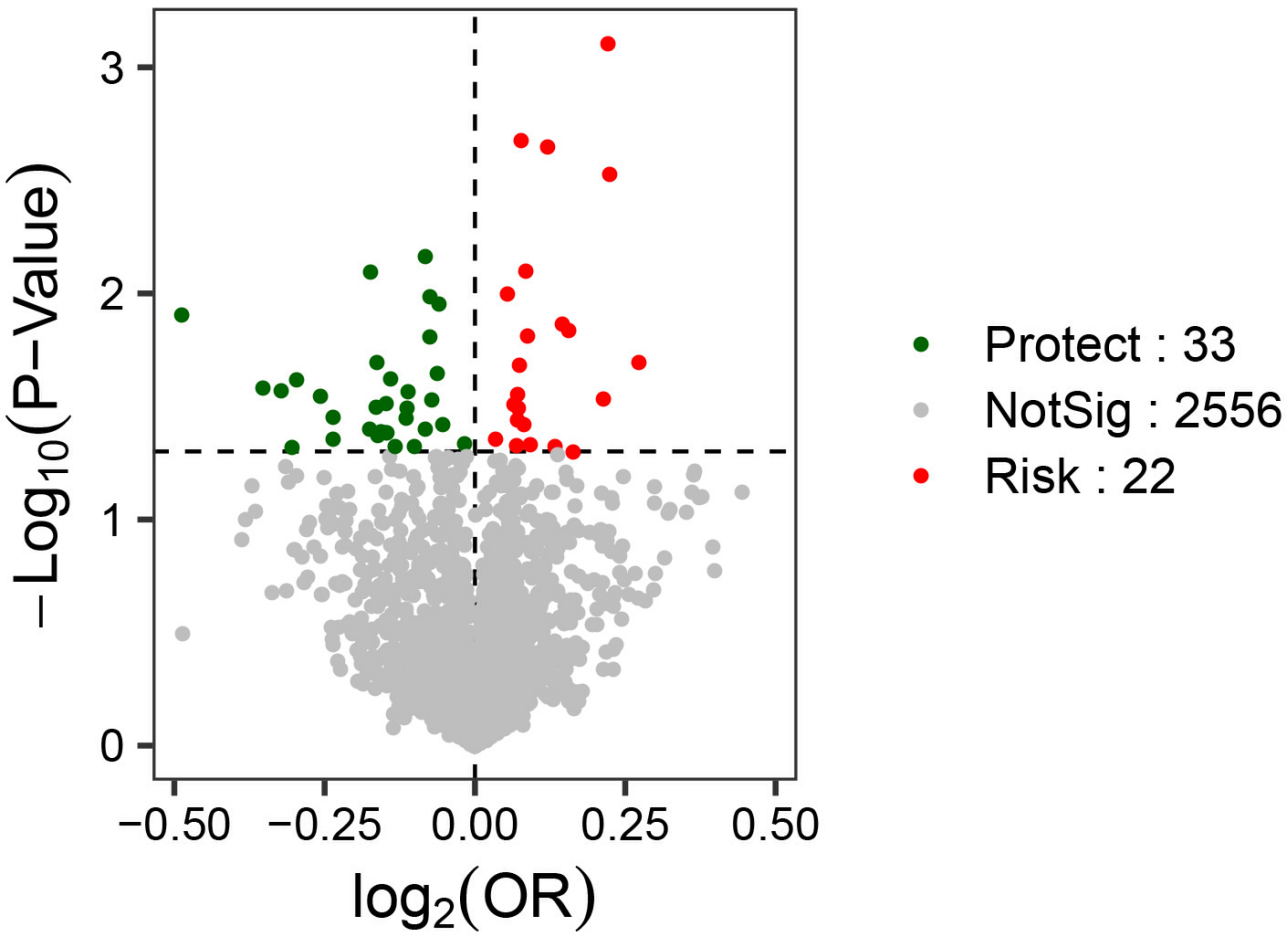
**Volcano plots of the causal associations among the gut microbiota, plasma metabolites, immune cells, blood cells, cytokines and schizophrenia (repeat the queue analysis results)**. The horizontal axis represents the magnitude of OR change (log_2_OR), and the vertical axis represents statistical significance (the log-transformed *p*).

We employed SCZ GWAS data from FinnGen for validation in the replication cohort. The IVs causally related to SCZ were gut microbiota, plasma metabolites and immune cells (Fig. 4) (**Supplementary Table 2–S1, S3 and S5**). In this replicated data, the trend was similar to that of the analytical results of the experimental cohort, and the most significant causal association with SCZ was ‘Francisellaceae abundance in stool’ within gut microbiota. We acquired 22 traits with risk (Risk) and 33 traits with protection (Protect) for SCZ in the two-sample MR analysis based on gut microbiota, plasma metabolites, immune cells and SCZ (Fig. 5). Reverse MR analysis was consistent with the outcomes of the experimental cohort, revealing the causal connection between plasma metabolites and immune cells and gut microbiota (**Supplementary Table 2–S7**). In the MVMR analysis of the replication cohort, six plasma metabolite traits were identified to play significant mediating roles in the causal association between gut microbiota and SCZ (**Supplementary Table 2–S9**). In this mediation analysis, the two causal pathways with the highest mediation proportions were both mediated by L-carnitine levels, accounting for 11.80% and 12.68% (**Supplementary Table 2–S11**). All the aforementioned correlation analyses underwent sensitivity analyses to ensure the reliability of the analytical results (**Supplementary Table 2–S2, S4, S6, S8, S10 and S12**).

## Discussion

Given the central role of the brain–gut axis as a bidirectional communication network connecting the central nervous system and the gastrointestinal tract via neural, endocrine and immune pathways [43], this study employed MR to systematically investigate the potential causal link between gut microbiota and SCZ. To gain comprehensive insight into this intricate interplay, we extended our analysis beyond gut microbial influences by incorporating plasma metabolites, which represent endocrine signalling, and peripheral immune cells as key mediating factors. Additionally, blood cell profiles and cytokines were included to capture immune-related dynamics [44]. By applying a mediation MR framework, we aimed to clarify the specific causal pathways through which gut microbiota alterations may contribute to SCZ development via metabolic and immune mediators. This analytical approach not only enhances our understanding of the brain–gut axis in SCZ aetiology but also offers a foundation for future therapeutic interventions targeting gut microbial modulation.

In this study, our primary task was to explore the potential etiological factors of SCZ by conducting TSTR analysis. This analysis constituted the first part of our research, wherein SCZ was designated as the outcome variable, and a series of biological traits, including the gut microbiota and other traits, was designated as exposure factors to reveal their potential correlations with SCZ. Notably, in this work, we found a significant positive causal association between the gut microbiota and SCZ. Among the numerous gut microbiota traits related to SCZ, two microbial groups were particularly prominent, namely, *Paenibacillales* and *Acetobacterales*. Specifically, *Paenibacillales* belongs to the Bacilli class under the Firmicutes phylum, whereas *Acetobacterales* belongs to the *Alphaproteobacteria* class under the Proteobacteria phylum. The specific abundance variations of these two microbial groups in the gut microbiota seem to be associated with the risk of SCZ onset. Notably, when reviewing relevant literature, we found a previous study on breast cancer that also involved *Paenibacillales*. This study indicated that the enrichment of *Paenibacillales* significantly increases the risk of granulocytopenia [45]. This result not only reinforces the important role of *Paenibacillales* in human health but also offers novel insights into the intricate interplay between gut microbiota and the host immune system. Additionally, the findings of this breast cancer study provide strong support for our current research, demonstrating a close interrelationship between the gut microbiota and immune cells and laying a solid foundation for our subsequent mediator MR analysis.

Prior to commencing mediation analysis, the research team carried out independent MR analyses on cytokines, gut microbiota, immune cells, blood cells and plasma metabolomics. The central aim of this portion of the analysis was to identify mediator variables that can exert bidirectional mediating effects within the cascading pathway between gut microbiota and SCZ (i.e., the brain–gut axis regulatory pathway) from the abovementioned variables demonstrated to have a causal association with SCZ. Through statistical testing and result screening, we determined 9 statistically significant associative relationships between plasma metabolomics and gut microbiota. By contrast, we found 2 distinct associative signals between immune cells and gut microbiota. A total of these 11 valid associative outcomes will be used as crucial fundamental data in the subsequent MVMR analysis stage.

In the MVMR analysis of this research, gut microbiota traits were regarded as the exposure factors, SCZ was used as the outcome variable and the plasma metabolites and immune cell traits screened through reverse MR analysis were considered mediator variables. The results of MVMR analysis revealed several significant discoveries. Specifically, after adjusting for the influence of gut microbiota, seven plasma metabolite traits and two immune cell traits exhibited significant causal associations with SCZ. Thus, we successfully determined nine mediating relationships between gut microbiota and SCZ, encompassing seven causal pathways mediated by plasma metabolites and two causal pathways mediated by immune cells. The majority of these causal pathways demonstrated a high degree of consistency in the direction of their total effects, indirect effects and direct effects. Among these causal pathways, we focused on the most negatively mediating potential pathway, namely, the pathway from CAG-83 sp000435555 to SCZ mediated by CD8 on naive CD8+ T cells. CAG-83 sp000435555 is an anaerobic bacterium isolated from the human gut, classified within the family *Acutalibacteraceae* (class Clostridia, phylum Firmicutes). This bacterium is involved in intestinal energy metabolism, with established associations with gut immunity and inflammation. Notably, it can ferment dietary fibre and generate SCFAs [46, 47]. It has been demonstrated that intestinal SCFAs increase the abundance of CD8+ T cells and enhance their cytotoxicity via the GPR109A/HOPX signaling pathway [48]. Simultaneously, antipsychotic agents (e.g., amisulpride) have been shown to modulate the gut microbiota composition in patients, specifically enriching the abundance of SCFA-producing bacterial taxa [49]. These findings provide experimental validation for the naive CD8+ T cell-mediated mediating pathway linking CAG-83 to SCZ, thereby supporting the validity of the present mediation analysis. Other outcomes of this mediation analysis can be analogically extended to propose additional potential novel directions for research into the mechanisms underlying SCZ.

A growing accumulation of research findings supports the critical mediating function of plasma metabolites and immune cells in the causal mechanism by which gut microbiota influences the development of SCZ. Numerous studies have conclusively demonstrated that an imbalance in the gut microbiota can disrupt the tryptophan metabolic pathway. For example, it may lead to abnormally elevated levels of kynurenine, thereby interfering with neurotransmitter homeostasis and neuroimmune regulation [9]. Moreover, this disruption allows the gut microbiota to affect the immune response mechanism of the central nervous system and the activation state of microglia via the blood–brain barrier permeation or the vagus nerve conduction pathways. This subsequently exacerbates the neuroinflammatory response and ultimately contributes to the pathophysiological progression of SCZ [50]. Clinical investigations have further verified that patients with SCZ exhibit unique characteristics of a disordered ‘microbiota–metabolite–immune’ network. On the one hand, this is evidenced by the excessive buildup of pro-inflammatory metabolites and a relative scarcity of anti-inflammatory metabolites. On the other hand, the changes in abundance of certain intestinal bacteria exhibit a significant correlation with the levels of peripheral cytokines and the severity of clinical symptoms [51]. Multi-omics integration analyses have shown the molecular mechanisms by which the gut microbiota indirectly regulates neurochemical functions through metabolic products and immune mediators. This has provided a theoretical foundation for intervention strategies targeting the gut microbiota in the context of SCZ [13]. Notwithstanding these findings, the precise causal pathways within this regulatory network remain unclear. Therefore, there is an urgent need for an increased number of high-quality experimental studies to validate and elucidate these mechanisms.

Although this study has yielded relevant findings in the target domain, several limitations persist in terms of its research design and data utilisation. Firstly, the GWAS data upon which this study relies exhibit notable high heterogeneity. This heterogeneity is primarily evident in the inevitable sample overlap and the variations in core characteristics such as genetic backgrounds and phenotypic distributions among the populations included in different datasets. This heterogeneity warrants caution when generalising the causal inference conclusions derived from this study to ethnic groups not included in the research. Otherwise, it may lead to biases in the applicability of the conclusions. Secondly, the intrinsic flaws of GWAS data and the limitations of external data support may also compromise the credibility of the study’s statistical outcomes. Finally, in terms of its methodological essence, the core value of MR as a causal inference method based on genetic variations is its ability to provide statistical evidence for causal associations, rather than elucidating specific biological mechanisms. Therefore, when determining the causal direction of the study findings or interpreting their clinical translational implications, a comprehensive analysis must be conducted by integrating multi-dimensional empirical evidence from various sources, such as molecular biology experiments and clinical cohort validations. Relying solely on the results of MR analysis may lead to a partial understanding of the causal relationship, complicating the thorough elucidation of its biological significance and therapeutic applicability.

In summary, through MVMR analysis, this study revealed various mediating relationships between gut microbiota traits and SCZ, including causal pathways mediated by plasma metabolites and immune cells. These findings offer novel perspectives and indicators for understanding the pathogenesis of SCZ and may also provide potential targets for future research and treatment.

## Conclusions

This MR study has unveiled causal associations between SCZ and multi-dimensional biomarkers. Specifically, these biomarkers encompass 11 gut microbiota, 35 plasma metabolites, 14 immune cells, 1 blood cell and 1 cytokine. Of these, the causal link between gut microbiota features and SCZ demonstrates the strongest effect size. Furthermore, through causal mediation analysis, this study has identified nine potential mediating pathways. In these pathways, seven plasma metabolites and two immune cells act as core mediating factors, functioning as a bridge in the causal link between gut microbiota and SCZ. This has elucidated a specific causal transmission pathway of “gut microbiota–mediating factors–SCZ”.

From genetic and molecular epidemiological perspectives, the aforementioned results have strengthened the evidence supporting the critical involvement of the brain–gut axis in the development of SCZ. This provides a theoretical foundation and potential targets for the subsequent development of prevention and treatment strategies for SCZ, which are based on gut microbiota regulation, metabolic intervention and immune modulation.

## Availability of Data and Materials

All the GWAS summary statistics employed in this research were derived from the GWAS Catalog (https://www.ebi.ac.uk/gwas/) and the University of Bristol (https://research-information.bris.ac.uk/en/datasets/). The accession numbers of all GWAS data are consolidated in (**Supplementary Table 1–S1**).

## Supplementary Material

Supplementary material associated with this article can be found, in the online version, at https://doi.org/10.62641/aep.v54i1.2014.

## References

[1] American Psychiatric Association, DSM-5 Task Force (2013). Diagnostic and statistical manual of mental disorders: DSM-5™ (5th ed.).

[2] Jonas K, Lian W, Callahan J, Ruggero CJ, Clouston S, Reichenberg A (2022). The Course of General Cognitive Ability in Individuals With Psychotic Disorders. *JAMA Psychiatry*.

[3] Javitt DC (2023). Cognitive Impairment Associated with Schizophrenia: From Pathophysiology to Treatment. *Annual Review of Pharmacology and Toxicology*.

[4] Gebreegziabhere Y, Habatmu K, Mihretu A, Cella M, Alem A (2022). Cognitive impairment in people with schizophrenia: an umbrella review. *European Archives of Psychiatry and Clinical Neuroscience*.

[5] Chen Y, Xu J, Chen Y (2021). Regulation of Neurotransmitters by the Gut Microbiota and Effects on Cognition in Neurological Disorders. *Nutrients*.

[6] Wang X, Wang Z, Cao J, Dong Y, Chen Y (2023). Gut microbiota-derived metabolites mediate the neuroprotective effect of melatonin in cognitive impairment induced by sleep deprivation. *Microbiome*.

[7] Alam A, Hana Z, Jin Z, Suen KC, Ma D (2018). Surgery, neuroinflammation and cognitive impairment. *EBioMedicine*.

[8] Cryan JF, O’Riordan KJ, Cowan CSM, Sandhu KV, Bastiaanssen TFS, Boehme M (2019). The Microbiota-Gut-Brain Axis. *Physiological Reviews*.

[9] Zhu F, Ju Y, Wang W, Wang Q, Guo R, Ma Q (2020). Metagenome-wide association of gut microbiome features for schizophrenia. *Nature Communications*.

[10] Genedi M, Janmaat IE, Haarman BBCM, Sommer IEC (2019). Dysregulation of the gut-brain axis in schizophrenia and bipolar disorder: probiotic supplementation as a supportive treatment in psychiatric disorders. *Current Opinion in Psychiatry*.

[11] Ju S, Shin Y, Han S, Kwon J, Choi TG, Kang I (2023). The Gut-Brain Axis in Schizophrenia: The Implications of the Gut Microbiome and SCFA Production. *Nutrients*.

[12] Rogers GB, Keating DJ, Young RL, Wong ML, Licinio J, Wesselingh S (2016). From gut dysbiosis to altered brain function and mental illness: mechanisms and pathways. *Molecular Psychiatry*.

[13] Fan Y, Gao Y, Ma Q, Yang Z, Zhao B, He X (2022). Multi-Omics Analysis Reveals Aberrant Gut-Metabolome-Immune Network in Schizophrenia. *Frontiers in Immunology*.

[14] Nakahara T, Tsugawa S, Noda Y, Ueno F, Honda S, Kinjo M (2022). Glutamatergic and GABAergic metabolite levels in schizophrenia-spectrum disorders: a meta-analysis of 1H-magnetic resonance spectroscopy studies. *Molecular Psychiatry*.

[15] Wang Z, Yuan X, Zhu Z, Pang L, Ding S, Li X (2024). Multiomics Analyses Reveal Microbiome-Gut-Brain Crosstalk Centered on Aberrant Gamma-Aminobutyric Acid and Tryptophan Metabolism in Drug-Naïve Patients with First-Episode Schizophrenia. *Schizophrenia Bulletin*.

[16] Wu D, Wu Q, Li F, Wang Y, Zeng J, Tang B (2024). Free water alterations in different inflammatory subgroups in schizophrenia. *Brain, Behavior, and Immunity*.

[17] Gül Çakıl A, Kaya H, Sakallı Nural A, Çakmak IB, Okay İT, Göka E (2023). Neutrophil gelatinase-associated lipocalin (NGAL) and tumor necrosis factor-α (TNF-α) levels in patients with schizophrenia. *Psychopharmacology*.

[18] Halstead S, Siskind D, Amft M, Wagner E, Yakimov V, Shih-Jung Liu Z (2023). Alteration patterns of peripheral concentrations of cytokines and associated inflammatory proteins in acute and chronic stages of schizophrenia: a systematic review and network meta-analysis. *The Lancet. Psychiatry*.

[19] Debnath M, Berk M (2017). Functional Implications of the IL-23/IL-17 Immune Axis in Schizophrenia. *Molecular Neurobiology*.

[20] Ermakov EA, Mednova IA, Boiko AS, Buneva VN, Ivanova SA (2023). Chemokine Dysregulation and Neuroinflammation in Schizophrenia: A Systematic Review. *International Journal of Molecular Sciences*.

[21] Sekar A, Bialas AR, de Rivera H, Davis A, Hammond TR, Kamitaki N (2016). Schizophrenia risk from complex variation of complement component 4. *Nature*.

[22] Yilmaz M, Yalcin E, Presumey J, Aw E, Ma M, Whelan CW (2021). Overexpression of schizophrenia susceptibility factor human complement C4A promotes excessive synaptic loss and behavioral changes in mice. *Nature Neuroscience*.

[23] Zhu Y, Webster MJ, Walker AK, Massa P, Middleton FA, Weickert CS (2023). Increased prefrontal cortical cells positive for macrophage/microglial marker CD163 along blood vessels characterizes a neuropathology of neuroinflammatory schizophrenia. *Brain, Behavior, and Immunity*.

[24] Reale M, Costantini E, Greig NH (2021). Cytokine Imbalance in Schizophrenia. From Research to Clinic: Potential Implications for Treatment. *Frontiers in Psychiatry*.

[25] Corsi-Zuelli F, Deakin B (2021). Impaired regulatory T cell control of astroglial overdrive and microglial pruning in schizophrenia. *Neuroscience and Biobehavioral Reviews*.

[26] Didelez V, Sheehan N (2007). Mendelian randomization as an instrumental variable approach to causal inference. *Statistical Methods in Medical Research*.

[27] Birney E (2022). Mendelian Randomization. *Cold Spring Harbor Perspectives in Medicine*.

[28] Carter AR, Sanderson E, Hammerton G, Richmond RC, Davey Smith G, Heron J (2021). Mendelian randomisation for mediation analysis: current methods and challenges for implementation. *European Journal of Epidemiology*.

[29] Ge L, Zhu L, Su C, Jin Z (2025). Exploring causal gut-brain axes in Alzheimer’s disease using mediation Mendelian randomization analysis. *Journal of Alzheimer’s Disease*.

[30] Zhang J, Fan W, Su C, Jin Z (2025). Influence of immune cells and inflammatory factors on Alzheimer’s disease axis: evidence from mediation Mendelian randomization study. *BMC Neurology*.

[31] Skrivankova VW, Richmond RC, Woolf BAR, Yarmolinsky J, Davies NM, Swanson SA (2021). Strengthening the Reporting of Observational Studies in Epidemiology Using Mendelian Randomization: The STROBE-MR Statement. *JAMA*.

[32] Qin Y, Havulinna AS, Liu Y, Jousilahti P, Ritchie SC, Tokolyi A (2022). Combined effects of host genetics and diet on human gut microbiota and incident disease in a single population cohort. *Nature Genetics*.

[33] Chen Y, Lu T, Pettersson-Kymmer U, Stewart ID, Butler-Laporte G, Nakanishi T (2023). Genomic atlas of the plasma metabolome prioritizes metabolites implicated in human diseases. *Nature Genetics*.

[34] Orrù V, Steri M, Sidore C, Marongiu M, Serra V, Olla S (2020). Complex genetic signatures in immune cells underlie autoimmunity and inform therapy. *Nature Genetics*.

[35] Vuckovic D, Bao EL, Akbari P, Lareau CA, Mousas A, Jiang T (2020). The Polygenic and Monogenic Basis of Blood Traits and Diseases. *Cell*.

[36] Ahola-Olli AV, Würtz P, Havulinna AS, Aalto K, Pitkänen N, Lehtimäki T (2017). Genome-wide Association Study Identifies 27 Loci Influencing Concentrations of Circulating Cytokines and Growth Factors. *American Journal of Human Genetics*.

[37] Sakaue S, Kanai M, Tanigawa Y, Karjalainen J, Kurki M, Koshiba S (2021). A cross-population atlas of genetic associations for 220 human phenotypes. *Nature Genetics*.

[38] Pierce BL, Ahsan H, Vanderweele TJ (2011). Power and instrument strength requirements for Mendelian randomization studies using multiple genetic variants. *International Journal of Epidemiology*.

[39] Verbanck M, Chen CY, Neale B, Do R (2018). Detection of widespread horizontal pleiotropy in causal relationships inferred from Mendelian randomization between complex traits and diseases. *Nature Genetics*.

[40] Gudicha DW, Schmittmann VD, Vermunt JK (2017). Statistical power of likelihood ratio and Wald tests in latent class models with covariates. *Behavior Research Methods*.

[41] Sanderson E (2021). Multivariable Mendelian Randomization and Mediation. *Cold Spring Harbor Perspectives in Medicine*.

[42] Tönnies T, Schlesinger S, Lang A, Kuss O (2023). Mediation Analysis in Medical Research. *Deutsches Arzteblatt International*.

[43] Socała K, Doboszewska U, Szopa A, Serefko A, Włodarczyk M, Zielińska A (2021). The role of microbiota-gut-brain axis in neuropsychiatric and neurological disorders. *Pharmacological Research*.

[44] Agirman G, Yu KB, Hsiao EY (2021). Signaling inflammation across the gut-brain axis. *Science*.

[45] Nguyen SM, Tran HTT, Long J, Shrubsole MJ, Cai H, Yang Y (2024). Gut microbiome in association with chemotherapy-induced toxicities among patients with breast cancer. *Cancer*.

[46] Park M, Almunawar SNA, Lim RRX, Haldar S, Henry CJ, Moskvin OV (2024). Genomic reconstruction and dietary response assessment of Three Clostridium leptum-related bacteria isolated from fecal samples of Singapore subjects. *bioRxiv*.

[47] Tan J, McKenzie C, Potamitis M, Thorburn AN, Mackay CR, Macia L (2014). The role of short-chain fatty acids in health and disease. *Advances in Immunology*.

[48] Yu X, Ou J, Wang L, Li Z, Ren Y, Xie L (2024). Gut microbiota modulate CD8+ T cell immunity in gastric cancer through Butyrate/GPR109A/HOPX. *Gut Microbes*.

[49] Zheng J, Lin Z, Ko CY, Xu JH, Lin Y, Wang J (2022). Analysis of Gut Microbiota in Patients with Exacerbated Symptoms of Schizophrenia following Therapy with Amisulpride: A Pilot Study. *Behavioural Neurology*.

[50] Zhou L, Wu Q, Jiang L, Rao J, Gao J, Zhao F (2025). Role of the microbiota in inflammation-related related psychiatric disorders. *Frontiers in Immunology*.

[51] Li S, Zhuo M, Huang X, Huang Y, Zhou J, Xiong D (2020). Altered gut microbiota associated with symptom severity in schizophrenia. *PeerJ*.

